# Blepharospasm

**Published:** 1892-01-23

**Authors:** 


### THE PRACTITIONER'S MIRROR AND RETROSPECT.

*The Editor will be glad to receive offers of co-operation and contributions from members of the profession. All letters should be addressed to* THE EDITOR, THE LODGE, PORCHESTER SQUARE, LONDON, W.]

### MEDICINE.

### BLEPHAROSPASM.

Blepharospasm, or spasmodic involuntary contraction of the orbicularis palpebrarum muscle, is a reflex act generally due to some irritation of the fifth nerve, and may be brought about by irritation of any terminal branch of that nerve, most commonly by ulcers of the cornea, herpes of the fifth nerve involving the cornea, ulcers in the nares or mouth, decayed teeth, &c. It is often accompanied by photophobia, in which case the light acting on the retina, the second nerve is the afferent nerve, and causes the ciliary muscle, through the third nerve, to contract; this drags on the corneal nerve-endings of the fifth nerve through which an afferent impulse is conveyed, and reflected to the seventh nerve, causes contraction of the orbicularis palpebrarum, and thus a double reflex act is performed. Blepharospasm may be clonic or tonic in character.

When clonic, nictitation occurs, a constant and repeated opening and closing of the eyelids, the act generally taking place in both eyes. This often occurs in high myopia, amblyopia from anæmia, or ciliary spasm, and capsular cataract; or it may be due to a kind of local chorea, or occur in nervous and hysterical subjects.

There is a slight form often experienced in persons who are over fatigued from any cause, or somewhat below par, as twitching, generally of the lower lid. The tonic spasm occurs commonly in children suffering from phlyctenular keratitis, or ulcers of the cornea; often in strumous subjects the eyelids are firmly closed and the patient is quite unable to open them. Any attempt to open them is accompanied by great pain, and it is often necessary to anæsthetize the patient before this can be done. The lids may appear somewhat swollen, and excoriated at the edges from the flow of tears over them, while at the outer canthus a fissure may be seen. When the lids are forced open, the under lid is often found to be curled inwards, with the eyelashes rubbing against the cornea. The spasm may be due to the impaction of a foreign body, such as a piece of steel, or to an insect having entered the conjunctival sac, but in these cases the lids can be separated usually with slight force, and everted without much difficulty. In the case of children just described, the upper lid will often evert, but without exposing any cornea, the everted eyelid showing much congestion of the veins. If the cause can be removed the spasm is easily cured, but generally the spasm must be overcome before anything can be done to remove the cause. The sudden application of cold, as by plunging the head into cold water, will sometimes succeed; the hypodermic injection of morphine may be tried.

The continuous current is employed by placing the positive pole behind the mastoid process, and passing the negative along the surface of the lid, and is sometimes very successful.

Blisters to the temporal region.

Division of the outer canthus with a pair of scissors, through its whole thickness, or of some fibres of the orbicularis, where the fissure is formed, will probably be followed by a free hæmorrhage from the engorged veins. When this has stopped, a pad moistened with cold lotion may be applied, and the patient put to bed in a darkened room. After a few hours the eye can generally be opened with little effort; the wound heals easily, and the muscle is not weakened.

The upper eyelid may be divided down the centre, and the two flaps turned back.

A simpler method is stretching of the orbicularis muscle by means of an ordinary spring speculum or pair of retractors, after full anæsthesia.

Blisters and local blood letting are sometimes successful in removing the spasm.

Division of the supra-orbital nerve has been performed for the same purpose. Sometimes it comes on after an operation, when it seems to be due to the pressure of the pad and bandage, and removal of these will often effect a cure, if not, the lid should be pulled down with the finger, and fixed to the cheek with adhesive plaister, or by the application of collodion flexile; as this dries, it contracts, and draws the lid away from the globe.

When the condition has lapsed into a chronic state, and treatment has failed to give satisfaction, a piece of skin may be removed from below the lid. An incision is made straight along just below the edge, and a curved incision joins its two extremities, the piece of skin is removed, and the edges united by sutures.

The mild form described as twitching of the muscles, of the lower lid requires nervine tonics—phosphorus, iron, and strychnine—and disappears as the health improves.

When due to errors of refraction, these must be corrected by suitable glasses.

